# Expression of epidermal growth factor, transforming growth factor-β1 and adiponectin in nipple aspirate fluid and plasma of pre and post-menopausal women

**DOI:** 10.1186/2050-7771-1-18

**Published:** 2013-04-15

**Authors:** Jessica A Miller, Patricia A Thompson, Iman A Hakim, Ana Maria Lopez, Cynthia A Thomson, Chiu-Hsieh Hsu, H-H Sherry Chow

**Affiliations:** 1 The University of Arizona Cancer Center, Tucson, AZ, USA; 2 Zuckerman College of Public Health, The University of Arizona, Tucson, AZ, USA

## Abstract

**Background:**

Nipple aspirate fluid (NAF) contains large amounts of protein thought to reflect the microenvironment of the breast, and is of interest in breast cancer prevention research. The correlation between specific NAF proteins to plasma concentrations have not been well studied in healthy women. We collected matched NAF and plasma from 43 healthy pre and postmenopausal women participating in an early phase clinical study to compare the levels of putative cancer protein biomarkers. We compared baseline NAF and plasma levels of epidermal growth factor (EGF), transforming growth factor-beta 1 (TGF-β1), and adiponectin and evaluated menopausal status and body mass index (BMI) as potential modifying factors.

**Findings:**

NAF and plasma levels of EGF, TGF-β1 and adiponectin were not correlated. EGF and TGF-β1 levels in NAF of premenopausal women were significantly higher than postmenopausal women (*P’s <* 0.01). These differences by menopausal status were not observed in plasma. Both NAF and plasma adiponectin levels were non-significantly higher in postmenopausal women. NAF biomarker levels were not associated with BMI whereas plasma EGF, TGF-β1 and adiponectin levels in postmenopausal women were all inversely correlated with BMI (*P*’s < 0.05).

**Conclusions:**

Protein biomarkers differ significantly between NAF and plasma and are affected differently by both BMI and menopausal status. This study demonstrates important differences in biological information gained by characterizing biomarkers in NAF compared to plasma and suggests each sample source may independently inform on breast cancer risk.

## Findings

### Introduction

Nipple aspirate fluid (NAF) is a rich source of protein postulated to more closely reflect the local breast microenvironment than plasma. Petrakis et al. [1] showed that simply producing NAF doubled a woman’s risk of breast cancer, which lead to a handful of studies using NAF as a source of breast tissue risk biomarkers [2-4]. The components of NAF are regularly metabolized and re-absorbed by the epithelial lining of the ductal/alveolar system [5]. It is hypothesized that drug, protein and hormone levels in NAF more closely reflects breast tissue environment and exposures than plasma [6,7]. In women with breast cancer, Kuerer et al. found no association between serum and NAF levels of soluble Her-2/*neu* although the Her-2/*neu* levels between the affected and unaffected breasts were highly correlated [8]. Whether protein levels in NAF of healthy women are correlated with plasma is largely unknown.

In an effort to better understand the relatedness between plasma and NAF composition in healthy women, we conducted a correlative analysis of candidate breast cancer risk markers in matched NAF and plasma samples from healthy pre- and postmenopausal women participating in a Phase I clinical trial. Here we report on our findings for biomarkers previously related to breast cancer, epidermal growth factor (EGF) [9], transforming growth factor-beta 1 (TGF-β1) [10], and adiponectin [11].

### Study participants

We recruited healthy women age 18–65 to a Phase I trial to evaluate the safety and feasibility of topical application of limonene as massage oil to the breast and to determine limonene levels in NAF. Details of the trial have been described elsewhere [12]. Participants were excluded if they were pregnant or breastfeeding, had invasive cancers within the past 5 years, participated in another clinical intervention trial within the past 3 months, had uncontrolled metabolic disorders, serious acute or chronic diseases, or were unable to produce NAF. The study was approved by the University of Arizona Human Subjects Committee and written consent was obtained from all participants.

### Sample collection

Baseline (pre-intervention) NAF was collected via breast massage and a Medela breast pump into capillary tubes and immediately diluted in phosphate buffered saline (1:10). Baseline blood sample was collected into Vacutainer tubes containing sodium heparin and centrifuged for plasma separation. Plasma and diluted NAF were stored at -80°C prior to analysis.

### Plasma and NAF biomarker analysis

NAF and plasma EGF, TGF-β1, and adiponectin were measured using ELISA based immunoassays (R&D Systems, Minneapolis, MN, USA). Plasma samples were diluted prior to the analysis according to the manufacturer instructions for EGF and TGF-β1, and were diluted 1:200 for the adiponectin assay. NAF samples were further diluted 1:100 – 400 for EGF, 1:20 – 40 for TGF-β1 and 1:3 – 1:20 for adiponectin. Assays were linear over the concentration range of 3.9 - 250 pg/mL, 31.2 – 2,000 pg/mL, and 3.9 - 250 ng/mL for EGF, TGF-β1, and adiponectin, respectively. Each sample was analyzed in duplicate for each assay (CVs <10% for all assays).

### Statistical analysis

Linear regression was performed to control for body mass index (BMI) while comparing differences between pre- and postmenopausal in protein biomarker levels in NAF as well as in plasma. Spearman correlation coefficients were derived to determine correlations between NAF and plasma biomarkers as well as BMI and NAF or plasma protein biomarkers. A *P*-value of < 0.05 was considered statistically significant.

### Participant characteristics

Matched baseline NAF and plasma samples were available from 16 premenopausal and 27 postmenopausal eligible women for the cross-sectional analysis; demographics are presented in Table 1.

**Table 1:** Participant demographics

	**Premenopausal women (N = 16)**	**Postmenopausal women (N = 27)**
Age	45 (41.4 ± 10.9)^a^	56.0 (56.2 ± 5.0)^a^
Body Mass Index (kg/m^2^)	25.3 (27.3 ± 6.0)^a^	25.1 (25.7 ± 4.6)^a^
Race/Ethnicity: *n* (%)		
Caucasian	15 (93.7)	24 (88.9)
Pacific Islander	1 (6.3)	0 (0.0)
Native American	1 (6.3)	1 (3.7)
Black	0 (0.0)	2 (7.4)

^a^median (mean ± SD).

### Plasma and NAF biomarker levels by menopausal status

**Figure 1:**
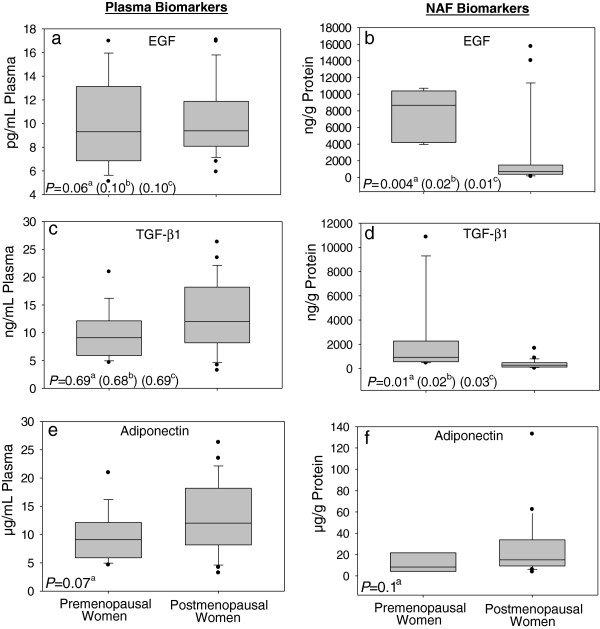
**Box plots illustrating differences between pre and postmenopausal women in plasma and NAF levels of Epidermal Growth Factor (EGF; a and b), Transforming Growth Factor Beta1 (TGF-β1; c and d) and Adiponectin (e and f). **^a^*P*-values derived from linear regression models with controlling for BMI. ^b^*P*-values derived from linear regression models with controlling for Adiponectin. ^c^*P*-values derived from linear regression models with controlling for BMI and Adiponectin.

Participants produced a wide range of NAF volume (3 – 50 μL) and in some participants there was insufficient NAF sample volume for all three biomarker measurements, yielding 33, 35 and 28 pairs of plasma and NAF measurements for EGF, TGF-β1 and adiponectin, respectively. The total protein concentration in NAF also varied widely among the study participants (13 – 101 mg/mL). Therefore, all NAF protein biomarker measurements were normalized by the total protein concentration. Figure 1 illustrates matched NAF and plasma biomarker levels by menopausal status (mean±SD presented in Additional file 1: Table S1). We corrected for BMI in all analyses. Postmenopausal women had significantly lower EGF (*P* = 0.004) and TGF-β1 (*P* = 0.01) levels in NAF when compared to premenopausal women. Plasma EGF levels were non-significantly higher in premenopausal women compared to postmenopausal while plasma TGF-β1 levels were not different (*P* = 0.06 and *P* = 0.69, respectively). Adiponectin levels were non-significantly higher in postmenopausal women in NAF (*P* = 0.15) as well as plasma (*P* = 0.07).

### Plasma and NAF biomarker correlations and relationship to BMI

None of the plasma biomarker levels were statistically significantly correlated to NAF levels (Table 2; Additional file 1: Figure S1). We also examined the correlation between levels of EGF, TGF-β1, and adiponectin and BMI by menopausal status (Table 3: Additional file 1: Figure S2). NAF biomarkers were not significantly correlated to BMI in either pre- or postmenopausal women. Plasma measures of EGF, TGF-β1 and adiponectin were all significantly inversely correlated with BMI in postmenopausal women (*P* = 0.003, *P* < 0.001, and *P* = 0.05; respectively). In premenopausal women, plasma adiponectin was significantly inversely associated with BMI (*P* = 0.02; n = 16). Analyses of the two-way interaction between BMI and menopausal status for plasma EGF and TGF-β1 concentrations were statistically significant (*P* = 0.04 and *P* = 0.01, respectively).

**Table 2:** Association between biomarker levels in NAF and plasma

	**Spearman correlation**	***P*-value**
**EGF**		
Premenopausal (n = 9)	−0.15	0.70
Postmenopausal (n = 24)	0.14	0.51
		
**TGF-**β**1**		
Premenopausal (n = 11)	−0.35	0.30
Postmenopausal (n = 24)	0.25	0.25
		
**Adiponectin**		
Premenopausal (n = 6)	−0.09	0.87
Postmenopausal (n = 22)	0.29	0.20

**Table 3:** Association between biomarker levels and BMI

**EGF**	**Spearman correlation**	***P*-value**
**NAF**		
Premenopausal (n = 9)	−0.20	0.61
Postmenopausal (n = 25)	0.06	0.79
**Plasma**		
Premenopausal (n = 16)	0.25	0.35
Postmenopausal (n = 27)	−0.55	0.003
**TGF-**β**1**		
**NAF**		
Premenopausal (n = 11)	−0.23	0.50
Postmenopausal (n = 26)	−0.09	0.68
**Plasma**		
Premenopausal (n = 16)	0.41	0.11
Postmenopausal (n = 27)	−0.60	<0.001
**Adiponectin**		
**NAF**		
Premenopausal (n = 6)	−0.31	0.54
Postmenopausal (n = 23)	0.15	0.50
**Plasma**		
Premenopausal (n = 16)	−0.59	0.02
Postmenopausal (n = 27)	−0.39	0.05

### Discussion

In a cohort of 43 healthy women, we measured the secreted protein biomarkers EGF, TGF-β1, and adiponectin in matched NAF and plasma samples. Interestingly, none of the three biomarkers in NAF were correlated to levels in plasma. Further, in postmenopausal women, levels of all three potential breast cancer biomarkers in plasma were inversely correlated with BMI, but there was no relationship between BMI and these biomarkers in NAF. With our small sample size, correlation coefficients ≥ 0.45 are necessary to determine statistically significant correlations between NAF and plasma or between biomarkers and BMI with 80% power. Our sample size, however, was sufficient to determine a statistically significant relationship between plasma adiponectin levels and BMI, a well-known association [13]. Therefore, this preliminary evidence suggests that NAF contains biological information distinct from plasma, despite the small sample size.

We are aware of only one other study reporting adiponectin levels in the NAF; Sauter *et al.* observed that 3 and 6 months after bariatric surgery, NAF adiponectin levels significantly increased in premenopausal women, but were unchanged in postmenopausal women [14]. Given the known inverse association between plasma adiponectin levels and BMI [15] and epidemiological evidence indicating an inverse relationship between adiponectin levels and breast cancer risk [11], it is interesting that NAF adiponectin levels in postmenopausal women appear to be independent of BMI in our work as well as that of Sauter *et al*.

To our knowledge, this is the first study to quantify TGF-β1 in NAF. Here TGF-β1 levels in NAF of premenopausal women were significantly higher than post-menopausal women, whereas plasma levels were similar between the two groups. These results likely reflect differential hormone levels in the breast by menopausal status.

NAF EGF levels in healthy, pre and postmenopausal women were similar to those previously reported [16-18]. Our finding of higher EGF levels in NAF of premenopausal women relative to postmenopausal women is consistent with a positive effect of sex hormones on EGF exposure in the breast [16]; as well as with other studies which have shown differences in plasma and NAF biomarkers by sex steroid hormone concentrations [19,20].

While our data suggests that circulating EGF, TGF-β1 and adiponectin in plasma are regulated differently from NAF protein levels, the main study limitation is the small sample size. Further, the premenopausal women enrolled in this study had, on average, a higher BMI compared to the postmenopausal women. This differs from the age expected BMI distribution [21], suggesting a participation bias. We attempted to adjust for this bias using adiponectin as a surrogate of metabolic disturbance [22]. After adjustment for BMI alone, adiponectin alone, or BMI and adiponectin, there were still significant differences in the NAF levels of the study proteins between pre and post-menopausal women, which were not observed in plasma. Reproductive variables such as time since lactation or last full birth may also affect NAF components [23], however, were not collected as part of the clinical study. Timing of sample collection for phase in the menstrual cycle poses another potential source of variation that was not controlled in this study. Chatterton *et al.*, however, has demonstrated that levels of NAF proteins including EGF, cathepsin D, and interleukin-6 were consistent throughout the phases of the menstrual cycle [24]. Therefore, it is likely that the variation in NAF protein levels in premenopausal women is a reflection of long-term hormonal (and environmental) exposures. Taken together, our findings suggest that the underlying biological factors associated with both BMI and menopausal status differentially influence protein expression in plasma as compared NAF. Future studies with larger sample sizes are warranted to develop the use of NAF as a minimally invasive strategy to identify risk factors and novel drug/prevention targets in the breast.

## Abbreviations

BMI: Body mass index; EGF: Epithelial growth factor; NAF: Nipple aspirate fluid; TGF-β1: Transforming growth factor beta 1; Her-2/neu: Human Epidermal Growth Factor Receptor 2; ELISA: Enzyme-linked immunosorbent assay.

## Competing interests

The authors’ declared that they have no competing interest.

## Authors’ contributions

JAM carried out the ELISA’s, drafted the manuscript, and contributed to the interpretation of the data. PAT helped to draft the manuscript and contributed to the interpretation of the data. IAH helped to draft the manuscript. AML helped to draft the manuscript. CAT helped to draft the manuscript. CHH carried out the statistical analysis. HSC conceived of the study design, was responsible for the coordination of the clinical trial, helped to draft the manuscript and contributed to the interpretation of the data. All authors have read and approved of the final manuscript.

## Supplementary Material

Additional file 1: Figure S1 Scatter plots illustrating null associations between plasma and NAF levels in premenopausal and postmonausal women of Epidermal Growth Factor (EGF; **a** and **b**), Transforming Growth Factor Beta 1 (TGF-β1; **c** and **d**) and Adiponectin (**e** and **f**). The appearance of an association between NAF and Plasma levels of TGF-β1 in postmenopausal women (**d**) seems to be driven by one woman with very high levels in both NAF and plasma. **Figure S2.** Scatter plots illustrating associations between BMI and plasma levels of Epidermal Growth Factor (EGF; **a**), Transforming Growth Factor Beta 1 (TGF-β1: **b**) and Adiponectin (**c**) in premenopausal women; between BMI and NAF levels of EGF (**d**) TGF-β1 (**e**) and Adiponectin (**f**) in premenopausal women; between BMI and plasma levels of EGF (**g**) TGF-β1 (**h**) and Adiponectin (**i**) in postmenopausal women; and between BMI and NAF levels of EGF (**j**) TGF-β1 (**k**) and Adiponectin (**l**) in postmenopausal women. **Table S1.** NAF and plasma biomarker levels. Click here for file
