# Supplementary material for: Expression of epidermal growth factor, transforming growth factor-β1 and adiponectin in nipple aspirate fluid and plasma of pre and post-menopausal women
Source: Biomark Res. 2013 Apr 15;1:18. doi: 10.1186/2050-7771-1-18 (PMC4177617; doi:10.1186/2050-7771-1-18)
Supplement: Additional file 1: Figure S1 — Scatter plots illustrating null associations between plasma and NAF levels in premenopausal and postmonausal women of Epidermal Growth Factor (EGF; a and b), Transforming Growth Factor Beta 1 (TGF-β1; c and d) and Adiponectin (e and f). The appearance of an association between NAF and Plasma levels of TGF-β1 in postmenopausal women (d) seems to be driven by one woman with very high levels in both NAF and plasma. Figure S2. Scatter plots illustrating associations between BMI and plasma levels of Epidermal Growth Factor (EGF; a), Transforming Growth Factor Beta 1 (TGF-β1: b) and Adiponectin (c) in premenopausal women; between BMI and NAF levels of EGF (d) TGF-β1 (e) and Adiponectin (f) in premenopausal women; between BMI and plasma levels of EGF (g) TGF-β1 (h) and Adiponectin (i) in postmenopausal women; and between BMI and NAF levels of EGF (j) TGF-β1 (k) and Adiponectin (l) in postmenopausal women. Table S1. NAF and plasma biomarker levels. [file 2050-7771-1-18-S1.pdf]

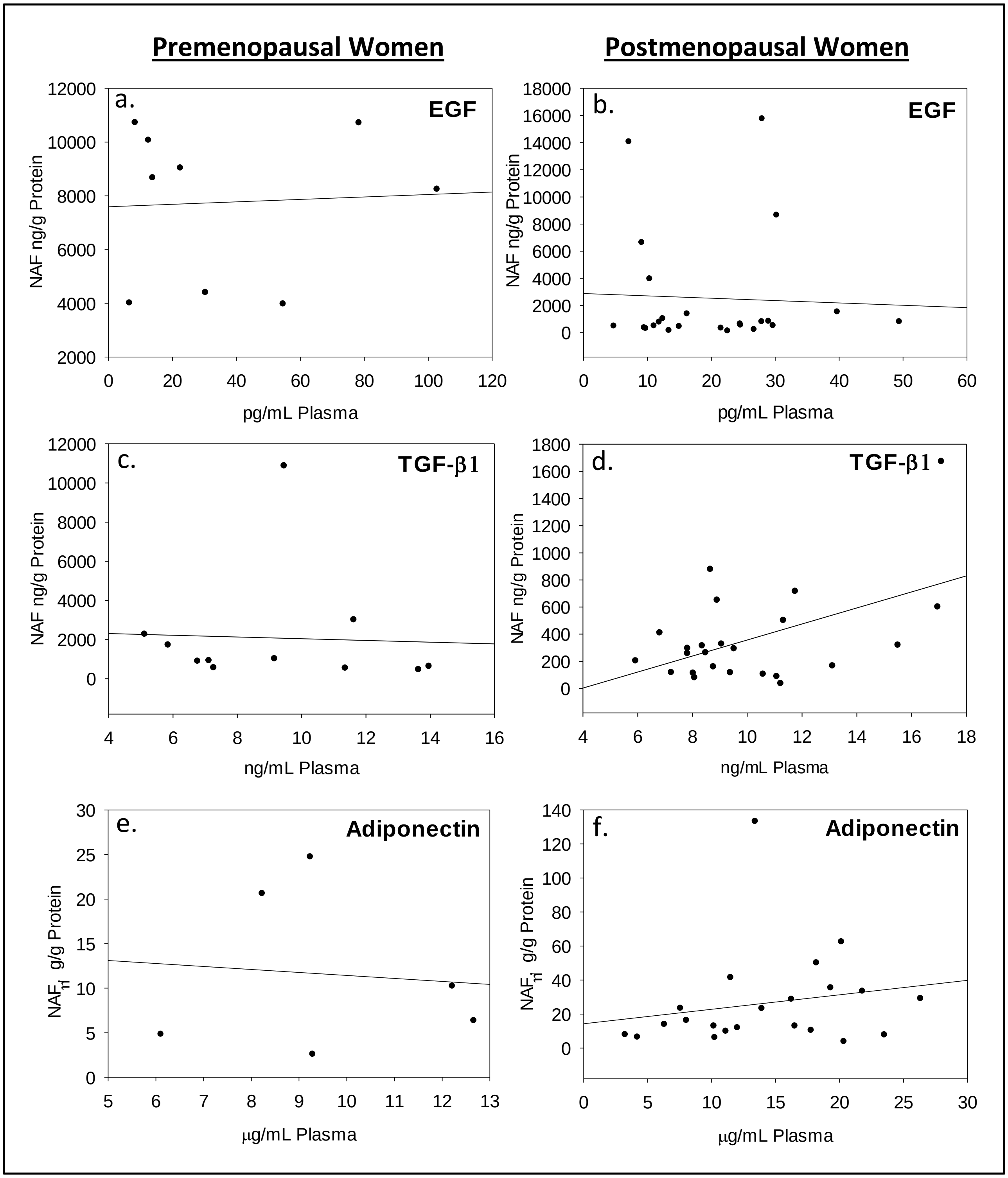

Supplementary Figure 1: Scatter plots illustrating null associations between plasma and NAF levels in premenopausal and postmonausal women of Epidermal Growth Factor (EGF; a and b), Transforming Growth Factor Beta 1 (TGF-β1; c and d) and Adiponectin (e and f). The appearance of an association between NAF and Plasma levels of TGF-β1 in postmenopausal women (d) seems to be driven by one woman with very high levels in both NAF and plasma.

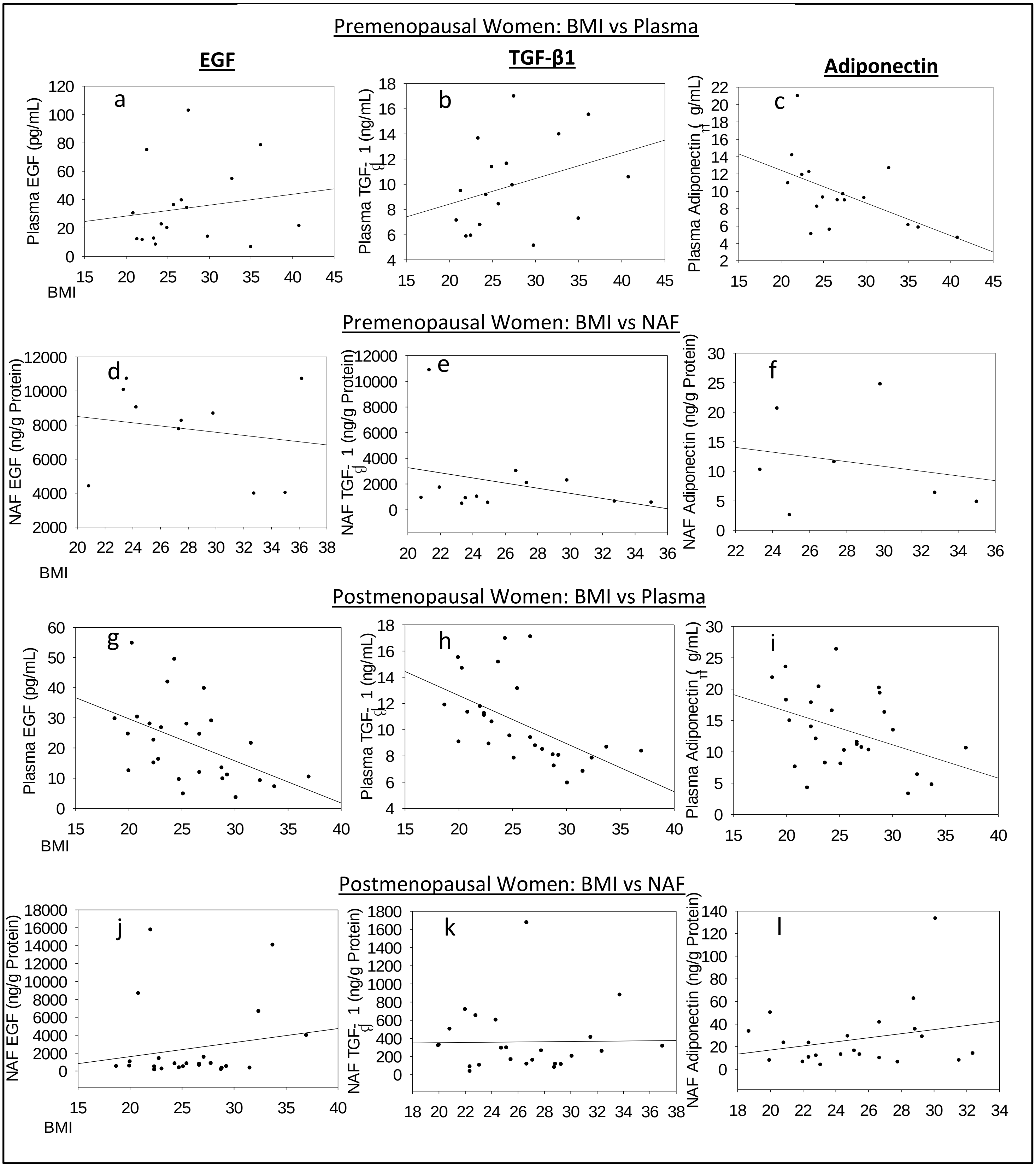

Supplementary Figure 2: Scatter plots illustrating associations between BMI and plasma levels of Epidermal Growth Factor (EGF; a), Transforming Growth Factor Beta 1 (TGF-β1: b) and Adiponectin (c) in premenopausal women; between BMI and NAF levels of EGF (d) TGF- β1 (e) and Adiponectin (f) in premenopausal women; between BMI and plasma levels of EGF (g) TGF- β1 (h) and Adiponectin (i) in postmenopausal women; and between BMI and NAF levels of EGF (j) TGF- β1 (k) and Adiponectin (l) in postmenopausal women.

Supplementary Table 1: NAF and plasma biomarker levels

**NAF Biomarker Levels**	**EGF (ng/g PT[a])**	**TGF-β1 (ng/g PT)**	**Adiponectin (μg/g PT)**
Premenopausal women	7,759 ± 2,854 [b] (n=9)	2,079 ± 3,027 (n=11)	11.6 ± 9.1 (n=6)
Postmenopausal women	2,532 ± 4,354 (n=24)	361 ± 357 (n=24)	26.3 ± 28.5 (n=22)
Pre vs postmenopausal women (*P*-value)	0.004[c] (0.02[d]) (0.01[e])	0.01 (0.02) (0.03)	0.15
**Plasma Biomarker Levels**	**EGF (pg/mL)**	**TGF-β (ng/mL)**	**Adiponectin (μg/mL)**
Premenopausal women (n=16)	34.1 ± 29.0[c]	9.92 ± 3.77	9.67 ± 4.19
Postmenopausal women (n=27)	21.6 ± 13.5	10.49 ± 3.13	13.37 ± 6.14
Pre vs postmenopausal women (*P*-value)	0.06[c] (0.10[d]) (0.10[e])	0.69 (0.68) (0.69)	0.07

[a]PT: protein
[b]mean ± SD
[c]*P*-values derived from linear regression models with controlling for BMI
[d]*P*-values derived from linear regression models with controlling for Adiponectin
[e]*P*-values derived from linear regression models with controlling for BMI and Adiponectin
